# Advanced exploitation of unmerged reflection data during processing and refinement with *autoPROC* and *BUSTER*

**DOI:** 10.1107/S2059798324001487

**Published:** 2024-02-27

**Authors:** Clemens Vonrhein, Claus Flensburg, Peter Keller, Rasmus Fogh, Andrew Sharff, Ian J. Tickle, Gérard Bricogne

**Affiliations:** a Global Phasing Ltd, Sheraton House, Castle Park, Cambridge, United Kingdom; ESRF, France

**Keywords:** unmerged reflection data, fitness analysis, reflection auditing, validation against diffraction data, radiation damage

## Abstract

The validation of structural models obtained by macromolecular X-ray crystallography against experimental diffraction data, whether before deposition into the PDB or after, is typically carried out exclusively against the merged data that are eventually archived along with the atomic coordinates. It is shown here that the availability of unmerged reflection data enables valuable additional analyses to be performed that yield improvements in the final models, and tools are presented to implement them, together with examples of the results to which they give access. The first example is the automatic identification and removal of image ranges affected by loss of crystal centering or by excessive decay of the diffraction pattern as a result of radiation damage. The second example is the ‘reflection-auditing’ process, whereby individual merged data items showing especially poor agreement with model predictions during refinement are investigated thanks to the specific metadata (such as image number and detector position) that are available for the corresponding unmerged data, potentially revealing previously undiagnosed instrumental, experimental or processing problems. The third example is the calculation of so-called *F*(early) − *F*(late) maps from carefully selected subsets of unmerged amplitude data, which can not only highlight the location and extent of radiation damage but can also provide guidance towards suitable fine-grained parametrizations to model the localized effects of such damage.

## Abstract 2

The final models for macromolecular X-ray crystallography studies are usually not only the result of refinement against some version of scaled and merged reflection data, but are often also analysed and validated purely against such merged data. Here, various examples are presented to show how the availability and use of unmerged reflection data can lead to better model analysis and improved model parametrization, as well as providing a path to better data processing and scaling.

## 1. Introduction

The mandatory deposition of experimental data (wwPDB, 2007 ▸) into the PDB archive (wwPDB Consortium, 2019 ▸) has enabled a wide variety of projects that make use of the original, processed and merged reflection data to not only provide better validation and potentially improved models, but also to drive method and software developments (Terwilliger & Bricogne, 2014 ▸). Recent work by the PDBx/mmCIF working group (Westbrook *et al.*, 2022 ▸) consolidated the description of unmerged reflection data to support richer descriptions and more useful depositions of scaled and unmerged reflection data (wwPDB, 2021 ▸).

Ultimately, better data might always be obtainable by performing a better X-ray diffraction experiment, either by (i) using a better data-collection protocol, detector or radiation source, (ii) optimizing an existing experimental setup and protocol or (iii) using improved crystal samples. When the opportunity for such a new experiment is not available, the first step in attempting an improvement in data quality often involves going back to the original raw diffraction data, *i.e.* the set of images containing raw diffraction counts in a two-dimensional pixel array. This is obviously only possible if these raw diffraction data are still available or have been deposited. As an intermediate stage between those two extremes – raw data at one end and the final merged reflection data (often structure-factor amplitudes and their standard uncertainties only) at the other – the unmerged reflection data in the form of individual measurements for a given unique reflection can serve as a useful compromise, provided that they carry sufficient metadata. It is the purpose of this article to illustrate the benefits that can be gained by exploiting various categories of such metadata through novel tools implemented in *autoPROC* (Vonrhein *et al.*, 2011 ▸) and *BUSTER* (Bricogne *et al.*, 2023 ▸).

## 2. Unmerged versus merged reflection data

Unmerged reflection data typically come in two flavours: (i) raw integrated intensities (and their standard uncertainty) with Lorentz and polarization corrections applied but before the scaling and outlier-rejection steps and (ii) intensities *I* and standard uncertainty σ after scaling and potential outlier rejection, *i.e.* just before the final merging step that produces merged intensities and their associated standard uncertainties.

In case (ii) the *N* individual scaled intensity measurements belonging to a given unique reflection are assumed to constitute a collection of independent measurements *I*_*i*_ of varying precision described by their respective variances *V*_*i*_ = 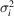 , having the same expectation value, namely the true value of the intensity for that unique reflection on the common scale of the *N* measurements. Under this assumption, the latter can then be combined (‘merged’) by inverse-variance-weighted averaging (https://en.wikipedia.org/wiki/Inverse-variance_weighting) to yield a maximum-likelihood estimator of the true intensity with improved precision.

In all modern merging procedures and programs, the merged intensity is therefore computed as 

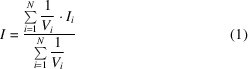

and the associated standard uncertainty as 

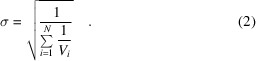




Besides their actual values used in this final merging step, raw integrated intensities are accompanied within the processing software by reflection attributes such as a symmetry flag indicating their relation to the unique reflection to which they belong, as well as the image number (or scan angle) and detector coordinates at which the corresponding spot centroids occur in the raw diffraction images. These attributes are available in some program-specific output files [for example INTEGRATE.HKL or XDS_ASCII.HKL from *XDS* (Kabsch, 2010 ▸) or the unmerged MTZ files used within the *CCP*4 suite (Agirre *et al.*, 2023 ▸)] but are lost once the scaled unmerged intensities are combined by inverse-variance-weighted averaging to produce the merged data that are then used for refinement and eventually deposited with the final refined model. Recent extensions to the PDB mmCIF dictionary allow these attributes to be deposited and archived as additional reflection items, thus giving access to remediation and improvement opportunities from such ‘enriched’ unmerged data, of which three examples are given in the following.

## 3. Benefits of using enriched unmerged data: principles

### 3.1. Unmerged reflection data: the image-fitness criterion

Robust automation of the processing of sets of diffraction images frequently requires the detection of poor image ranges, such as can result from the loss of centring during crystal rotation or from excessive radiation damage. For this purpose, a quantitative criterion is required to assess whether an image, or image range, is ‘fit to be retained’ in the final merging step or would best be excluded from it. Clearly, such a decision needs to consider the cost of such exclusions (especially in potentially compromising completeness) versus the benefits of removing particularly noisy contributors to the final merged intensities that may lead to certain data-quality metrics becoming unreliable.

Here, we introduce a new quantity that attempts to describe the contribution of a particular measurement *i* to the overall, inverse-variance-weighted merged intensity from *N* measurements for a given unique reflection. We first define a base quantity *S*_*i*_ as 

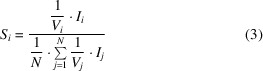

that describes the contribution of an individual measurement relative to the average contribution for a given unique reflection.

We then define the *fitness* of an image *j* as the average of the *S_i_* over all *n_j_* measurements associated with that image as 

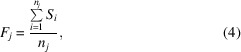

with the rationale that a low fitness value for an image indicates that it contains an accumulation of measurements with small relative contributions to the merged intensities of their respective unique reflections.

As such, it provides a criterion for identifying poor image ranges and can be used to automatically exclude them. This feature is available in *autoPROC*,^1^ where the fitness criterion is computed only over those unique reflections where the *I*/σ(*I*) value of the merged reflection is at least 2. The fitness values for each image are then analysed to detect consecutive images with values below a cutoff of, for example, 0.5, that are then automatically excluded in subsequent steps if they constitute sufficiently large ranges. The *I*/σ(*I*) threshold value (default 2) for selecting measurements over which to compute image fitness in the first place, as well as the cutoff value for the fitness below which images are excluded, can be adjusted by users of *autoPROC*. We have found in our regular analyses of large numbers of data sets that the current default values (i) include enough significant measurements to yield useful image fitness values and (ii) lead to rejection of only the most problematic image ranges.

The fitness criterion as a function of image number performs well in the detection and removal of poor image ranges associated, for example, with degradation or loss of crystal centring, as this causes an attenuation of the diffraction pattern. The raw intensity measurements extracted from these images will therefore need to be upscaled during subsequent processing to bring them onto the same scale as unattenuated equivalent measurements collected elsewhere in the data set. Upscaling an attenuated intensity by a factor *k* ≫ 1, and hence its variance by a factor of *k* ^2^, will decrease the quantity *S*_*i*_ in equation (3) by a factor of roughly 1/*k* ≪ 1, and therefore depress the fitness of images containing a systematic accumulation of such measurements. Although other factors may also be at play in lowering the fitness of an image or range of images, this connection between the upscaling of measurements from images affected by loss of centring and the decrease in the fitness of these images is a simple picture to keep in mind in rationalizing our definition and use of fitness.

The fitness criterion can also detect the effects of radiation damage, as these result in a similar attenuation of the diffraction pattern, but other metrics and tools might be better suited, or provide finer control, towards this specific task, for example the *R*_d_ metric (Diederichs, 2006 ▸) or the ΔCC_1/2_ metric (Assmann *et al.*, 2016 ▸).

### 3.2. Reflection auditing

By the end of the refinement process, our goal is to understand the reasons why certain reflections, or groups of reflections, have a low likelihood value given the final refined model. It is important to note that there will always be a few outlier reflections due to inherent errors in the data and the need for a balanced approach between the number of refined parameters, data quality and available restraints. However, identifying patterns of poorly fitting reflections can help to pinpoint specific issues in the data and potentially suggest improvements in data processing, experimental designs or sample quality.

After model refinement with *BUSTER*, the log(likelihood) value for each reflection given the current model is analysed, for example, as a function of resolution.^2^

The reflections with a very low log(likelihood) value are of particular interest here: we usually concentrate on those below 7σ [relative to the mean of all log(likelihood) values)], which would represent extreme outliers. Although such reflections could just be rejected automatically before any further refinement cycles (as is performed by default when using the *aB_autorefine* interface to *BUSTER*), we would like to also understand the underlying reasons and potential patterns leading to these outliers. The provided plots also include markers for known ice-ring resolution ranges, which can simplify the detection of ice contamination that might have affected the integration and/or scaling of the original reflection data. A similar capability to detect ice rings using the reflection data only in an analysis against resolution is available in the program *AUSPEX* (Thorn *et al.*, 2017 ▸).

#### 3.2.1. Looking at single reflections

Even if a small number of outlier reflections at the point of model refinement might seem to be nothing to be concerned about, if such outliers occur, for example, predominantly at the very low resolution end of the merged reflection data, they could have a much larger impact on both refinement performance and electron-density map quality than expected. The lowest resolution reflections are usually assumed to be both strong and with a relatively small associated σ value (standard uncertainty) and thus would carry a lot of weight during refinement and also dominate the computations dealing with the bulk-solvent contribution to the total structure-factor amplitude.

Low-resolution outliers are most commonly caused by an incorrect masking of the beamstop or beamstop holder during data processing. Automatic methods of masking shadowed static areas of the 2D diffraction images (such as the DEFPIX step in *XDS*) rely on a good contrast between the area of the detector receiving both diffraction and background scatter and the shadowed areas resulting from, for example, the beamstop and beamstop holder. With the usual modern data-collection strategies on pixel-array detectors of ‘low dose, high multiplicity and fine slicing’ (Mueller *et al.*, 2012 ▸), this contrast is not always present. Furthermore, beamstop holders come in a large variety of shapes and configurations that do not always adhere to the assumptions of such automatic tools (a circular beamstop centred on the direct beam position and on a simple, fairly rectangular beamstop holder).

#### 3.2.2. Looking at groups of reflections

If large sets of merged reflection data outliers are not grouped in an easily understood way, such as known ice-ring resolution ranges or very low resolution, it can be helpful to look for these outlier reflections in the context of the underlying unmerged reflection data. Such unmerged reflection data will typically carry additional information such as the rotation angle and detector position at which each measurement was originally recorded. This enables the visualization and analysis of these outliers as a function of rotation angle (*i.e.* image number) or detector position. The former can highlight particular image ranges that make a large contribution to the problematic (merged) reflection data, while the latter can help to identify potential detector or experimental setup problems such as damaged pixels or detector modules, incorrect handling of module gaps, dynamic shadowing due to goniostat movement not yet accounted for, *etc*.

### 3.3. Radiation-damage analysis via *F*(early) − *F*(late) maps

Any X-ray diffraction experiment will automatically cause radiation damage within the sample, mostly resulting in some breakdown of crystal order or site-specific damage at susceptible atoms and chemical groups (Garman & Weik, 2023 ▸). To help visualize the latter effects and to provide the additional information required for adequate parametrization of the model during refinement against the (dose-)averaged merged reflection data, we introduced so-called *F*(early) − *F*(late) (read: ‘early-minus-late’) maps into both our data-processing package *autoPROC* and the refinement suite *BUSTER*.^3^

These maps can be computed using the *F*(early) − *F*(late) difference as amplitude and the current best set of phases obtained during model refinement. They will show positive difference density where the ‘early’ data contains electrons while the ‘late’ data does not: this could be interpreted as a loss of electrons at this position, *i.e.* radiation damage. One will see negative values where additional electrons are present in the ‘late’ data set, which could be due to some movement of atoms, side chains or chemical groups and would usually be accompanied by some positive (loss of electrons) density close by.

#### 3.3.1. Creation of *F*(early) and *F*(late) amplitudes during data processing within *autoPROC*

**Figure 1:**
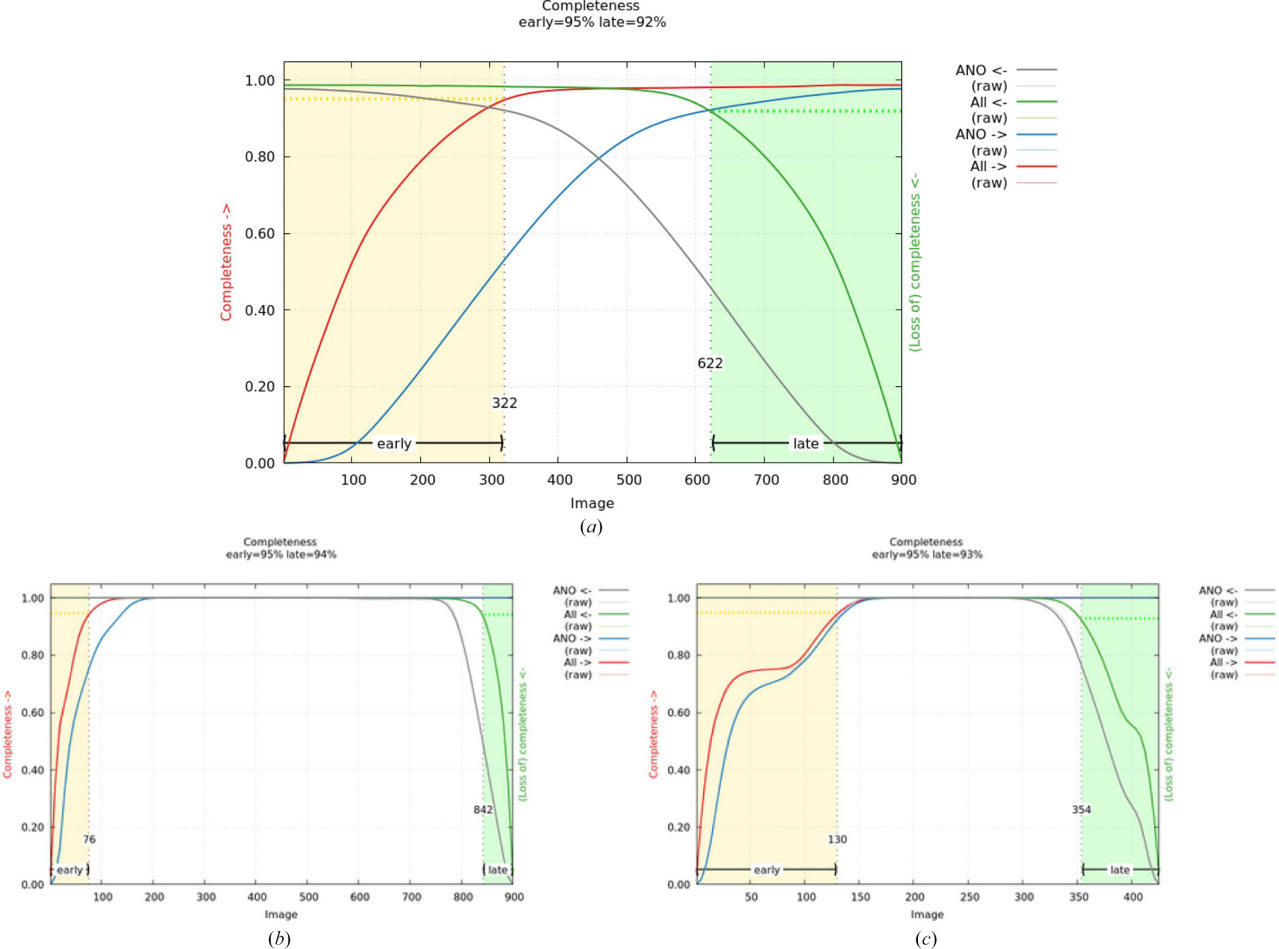
Cumulative completeness analysis to determine optimal segmentation for ‘early’ and ‘late’ data sets as performed by *autoPROC*. (*a*) PDB entry 5srx (Gahbauer *et al.*, 2023 ▸): the smoothed cumulative completeness as a function of increasing image number is given in red, while the associated anomalous completeness is given in blue; the related values as a function of decreasing image number are given in green and grey, respectively. The optimal image ranges to achieve high completeness, while at the same time maintaining a large distance (in image space), for the ‘early’ and ‘late’ data sets are marked by yellow and green backgrounds, respectively. (*b*) PDB entry 7kds (Seattle Structural Genomics Center for Infectious Disease, unpublished work): a high-symmetry space group (*P*4_1_32) together with a large rotation range during data collection (180°) achieves complete ‘early’ and ‘late’ data sets separated by a large image range (and therefore dose). (*c*) PDB entry 7wcj (Sharma *et al.*, 2022 ▸): the choice of an unfortunate starting angle for data collection shows as a plateauing of cumulative completeness at around image 50 (where a mirror plane in reciprocal space is crossed), leading to a slower increase of cumulative completeness while still accumulating dose.

During the scaling protocol of *autoPROC*, a final set of scale parameters is obtained from the full set of unmerged reflection data and, after scaling and merging, the cumulative completeness as a function of image number is computed in two ways: (i) ‘forward’ starting at the first image with increasing image number and (ii) ‘backward’ from the last image with decreasing image number. The values obtained by (i) can be used to define an ‘early’ image range that would give a reasonably complete set of merged reflections (compared to the maximum achievable completeness for a given data set), while the same can be performed using the data computed in (ii) for a set of images constituting the ‘late’ data set (see Fig. 1 ▸).

Because the goal is to have the largest possible distance (in terms of image numbers) between these two image ranges, the decision making as used in *autoPROC* involves a compromise between the completeness gained by adding more images to the set versus the reduction in gap width between the two sets. It is more important to maintain a sufficiently wide gap between the ‘early’ and ‘late’ data set to have a chance of visualizing radiation-damage effects than to gain a few more percentage points of completeness. This approach is very similar to that used by de Sanctis & Nanao (2012 ▸) in the context of experimental phasing making use of intensity differences due to site-specific radiation damage (see also Schiltz *et al.*, 2004 ▸).

Some important points to consider and remember are the following.

(i) The image number is used as a proxy for dose, with all of the simplifications that this entails. Ideally, the crystal would be fully bathed in the beam during the entire experiment and the flux would be constant throughout. This is a simplification that is nearly always necessary to make, since hardly any experiment will record this level of detailed information in the data available at the point of data processing, even if performed at a synchrotron beamline directly after the experiment has finished.

(ii) After selecting the appropriate image ranges, no further processing of the measurements contained within these image ranges is being performed. The scale as well as error model adjustment parameters previously determined for the full set of images are left unchanged, and the measurements are only merged into the two ‘early’ and ‘late’ data sets. This ensures that the two merged reflection data sets are scaled relative to each other as consistently as possible, or at least as consistently as the overall merged data used later for model refinement, and that the *F*(early) − *F*(late) difference we are interested in is as significant as possible.

#### 3.3.2. Use and analysis after refinement with *BUSTER*

When *BUSTER* (either the *refine* or *aB_autorefine* interface or when run as part of our *Pipedream* pipeline; Sharff *et al.*, 2023 ▸) is given an input MTZ file with data columns F_early/SIGF_early and F_late/SIGF_late (as, for example, produced automatically by *autoPROC* if at all possible), the final step will involve creating adequate map coefficients in the output MTZ file (F_early-late/PHI_early-late) and analysis of the *F*(early) − *F*(late) map against the current model. The former enables easy map calculation, for example in *Coot* (Emsley *et al.*, 2010 ▸), while the latter simplifies a quick overview regarding potential radiation damage at typical positions such as carboxyl groups (Glu/Asp side chains), S atoms (for example in Cys or Met) or halides (for example brominated ligands).

## 4. Benefits from using enriched unmerged data: examples

All examples are taken either from deposited PDB structures with available raw diffraction data collected on modern pixel-array detectors or recent PDB entries for which scaled and unmerged reflection data have been deposited.

### 4.1. Unmerged reflection data: the image-fitness criterion

**Figure 2:**
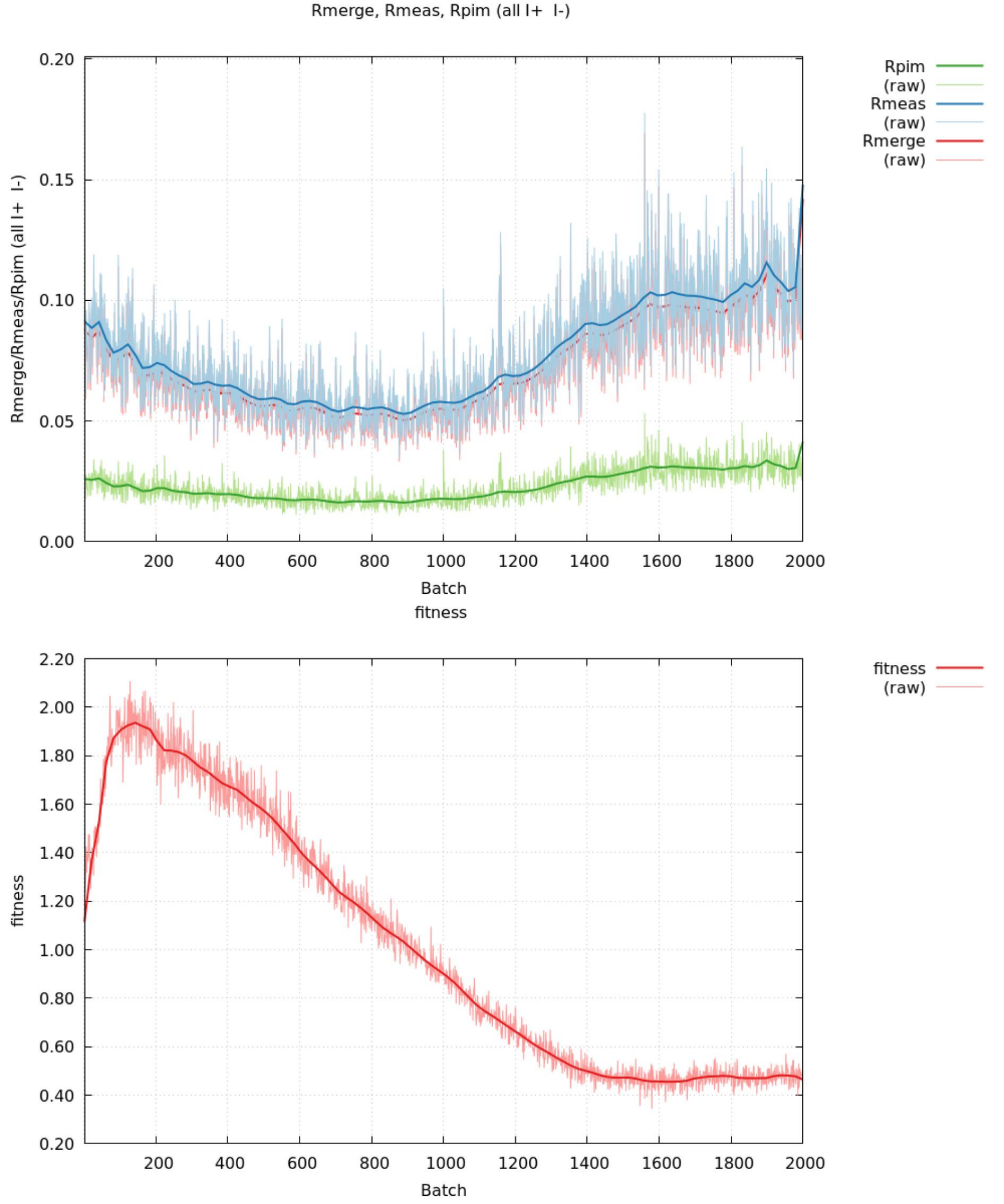
Merging *R* values (top, with *R*_p.i.m._ in green, *R*_meas_ in blue and *R*_merge_ in red) and fitness analysis (bottom) as a function of image number for the full set of deposited unmerged reflection data, *i.e.* 2000 images, for PDB entry 8aj2. The noisy raw data are shown as thin lines, while the smoothed values to be used in decision making are shown in bold. Plots were generated using *gnuplot* (Williams & Kelley, 2014 ▸) with Bezier curve smoothing as implemented therein.

The fitness criterion provides a robust method for, for example, automatically rejecting poor image ranges during data integration, scaling or merging. As an example, the deposited unmerged data for PDB entry 8aj2 (Batista *et al.*, 2023 ▸) can be used to analyse the effects of radiation damage by first calculating merging statistics for the full set of images (see Fig. 2 ▸). Based on a cutoff value of 0.5, images 1394–2000 should be excluded. The effects on the data-quality metrics are shown in Table 1 ▸: removal of 30% of the images has very little impact on completeness or outer-shell statistics (ignoring multiplicity-biased *R*_merge_ values). Only the overall 〈*I*/σ(*I*)〉 value shows a decrease when using the subset of available data. Performing an additional scaling using *AIMLESS* (Evans & Murshudov, 2013 ▸) on the full or restricted set of unmerged reflection data shows very similar data-quality metrics throughout: the overall 〈*I*/σ(*I*)〉 value for the subset has now increased. The potential impact on refinement performance and model quality still needs to be analysed (Diederichs & Karplus, 2013 ▸), ideally including a reprocessing of the full set of raw diffraction images so that the 30% poorer images towards the end of data collection could be excluded right from the start.

### 4.2. Reflection auditing

#### 4.2.1. Low-resolution data-processing problems

Apart from some obvious outliers within the highest resolution ice ring of PDB entry 5ofb (Douse *et al.*, 2018 ▸), a single reflection with Miller indices (1, 1, 0) at very low resolution shows a very low log(likelihood) value. This reflection was only ever measured once in the original experiment, which will not allow a comparison and possible outlier-rejection analysis, unless the intensity of this single measurement were to be compared with other measurements in a similar resolution range. However, at low resolution such comparisons become difficult, for example, due to questions of binning.

**Figure 3:**
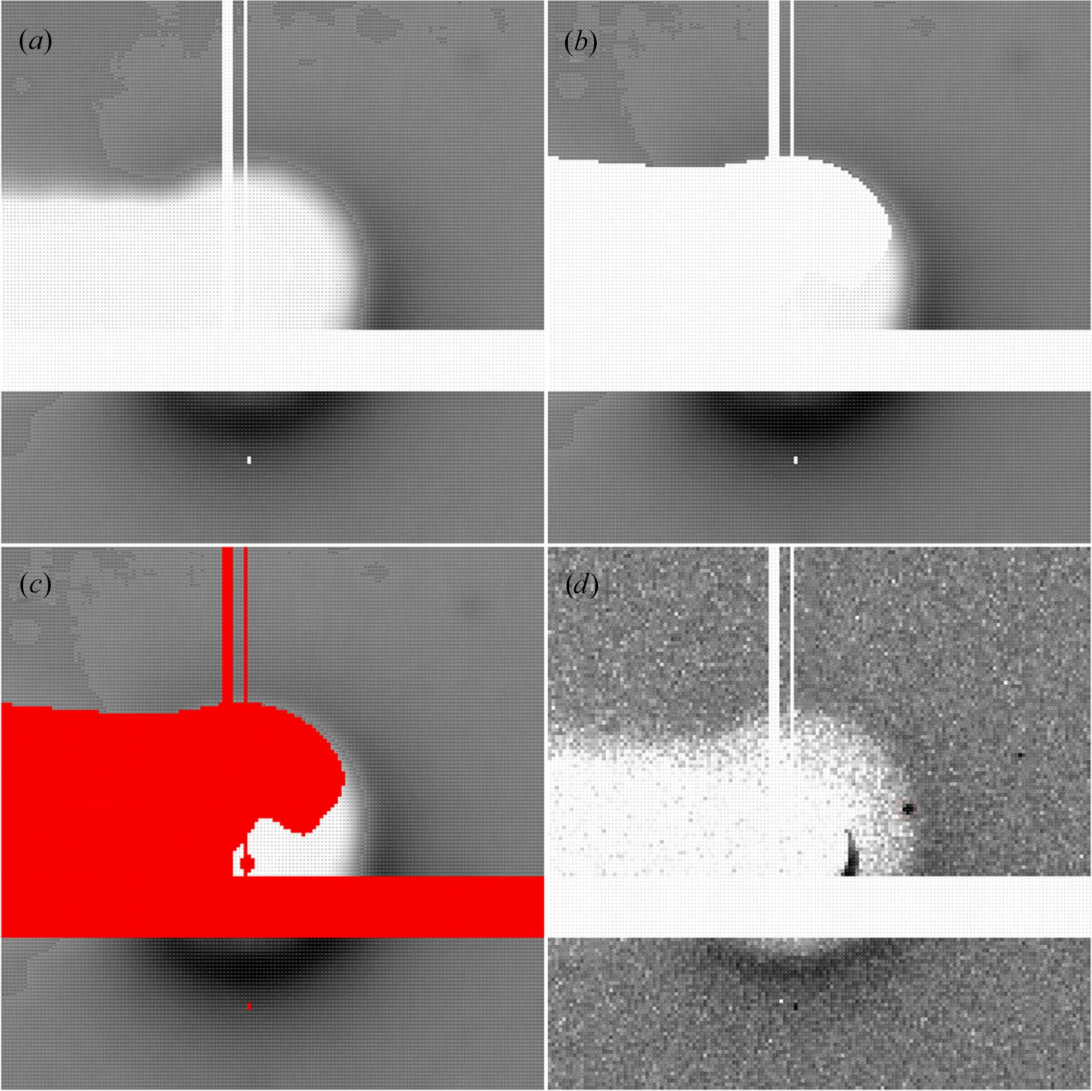
Low-resolution data problems for PDB entry 5ofb due to inadequate automatic beamstop masking. (*a*) Smoothed and averaged background scatter determined by the INIT step in *XDS*, (*b*) automatic beamstop masking as determined by the DEFPIX step in *XDS*, (*c*) individual pixels marked as inactive or masked and (*d*) reflection (1, 1, 0) partially obscured by the beamstop shadow in a region where the automatic masking procedure is underperforming.

The original diffraction spot sits very close to (or even within) the beamstop shadow and would not have been integrated if the beamstop had been manually masked during data processing. The particular shape of the beamstop, as well as its interaction with columns of damaged pixels and a module gap close by, make it difficult for any automatic masking procedure to correctly define the circular beamstop shape required for accurate masking at this point (see Fig. 3 ▸).

#### 4.2.2. Ice contamination

**Figure 4:**
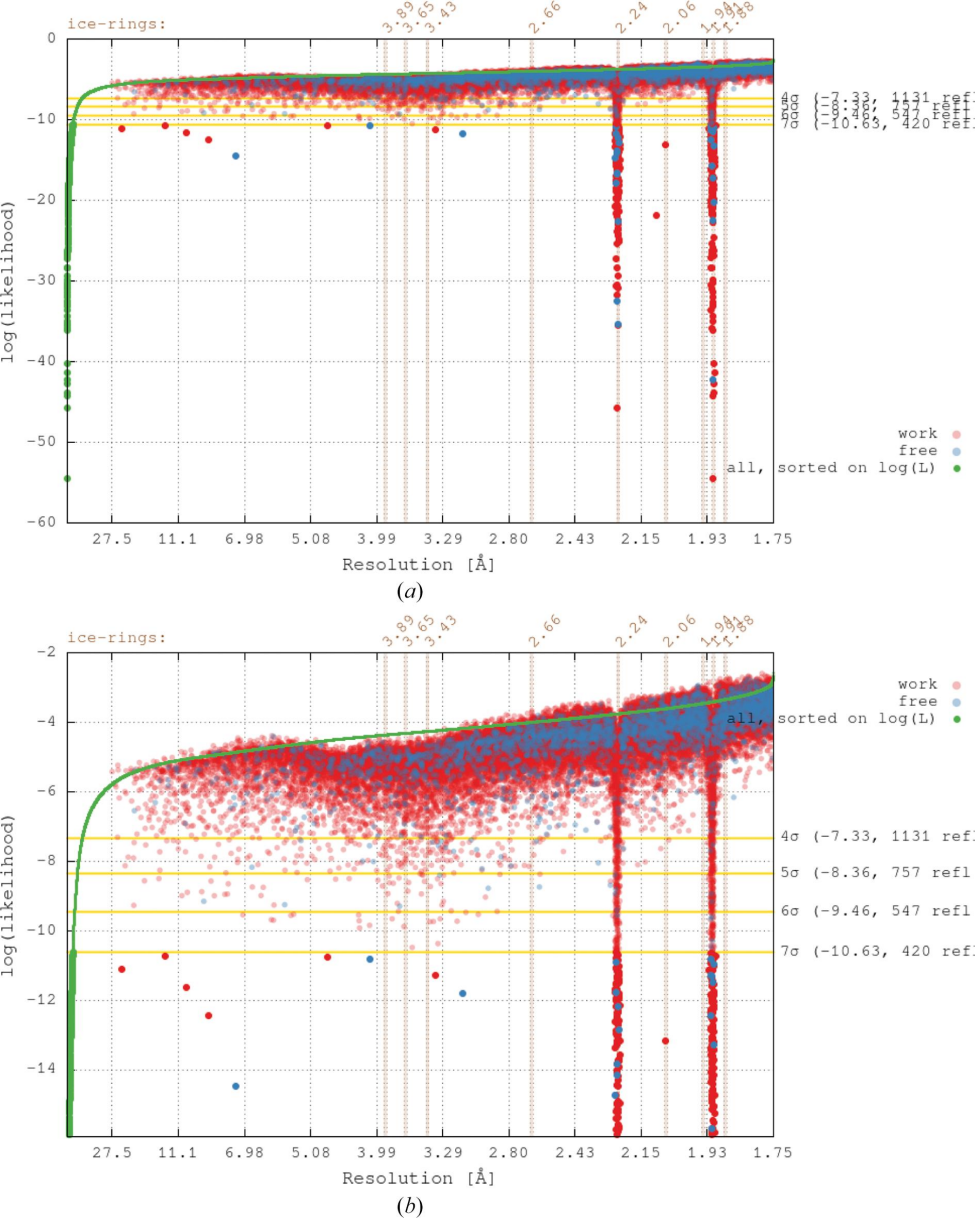
Log(likelihood) values of unique reflections after re-refinement of PDB entry 4z48 using *BUSTER*. Reflections in the working set are shown in red and those in the test set are shown in blue. Those reflections with log(likelihood) values above −7σ are shown as semi-transparent circles. The green points are log(likelihood) values of all reflections sorted on increasing value. (*a*) Full range of log(likelihood) values. (*b*) Close-up to highlight finer details of reflections with low log(likelihood) values.

Re-refinement of the deposited model for PDB entry 4z48 (Joint Center for Structural Genomics, unpublished work) with *BUSTER* (against the deposited amplitudes) followed by an analysis of per-reflection log(likelihood) values as a function of resolution shows a clear contamination of the deposited merged reflection data by ice rings present on the diffraction images (see Fig. 4 ▸).

#### 4.2.3. Problematic image ranges

**Figure 5:**
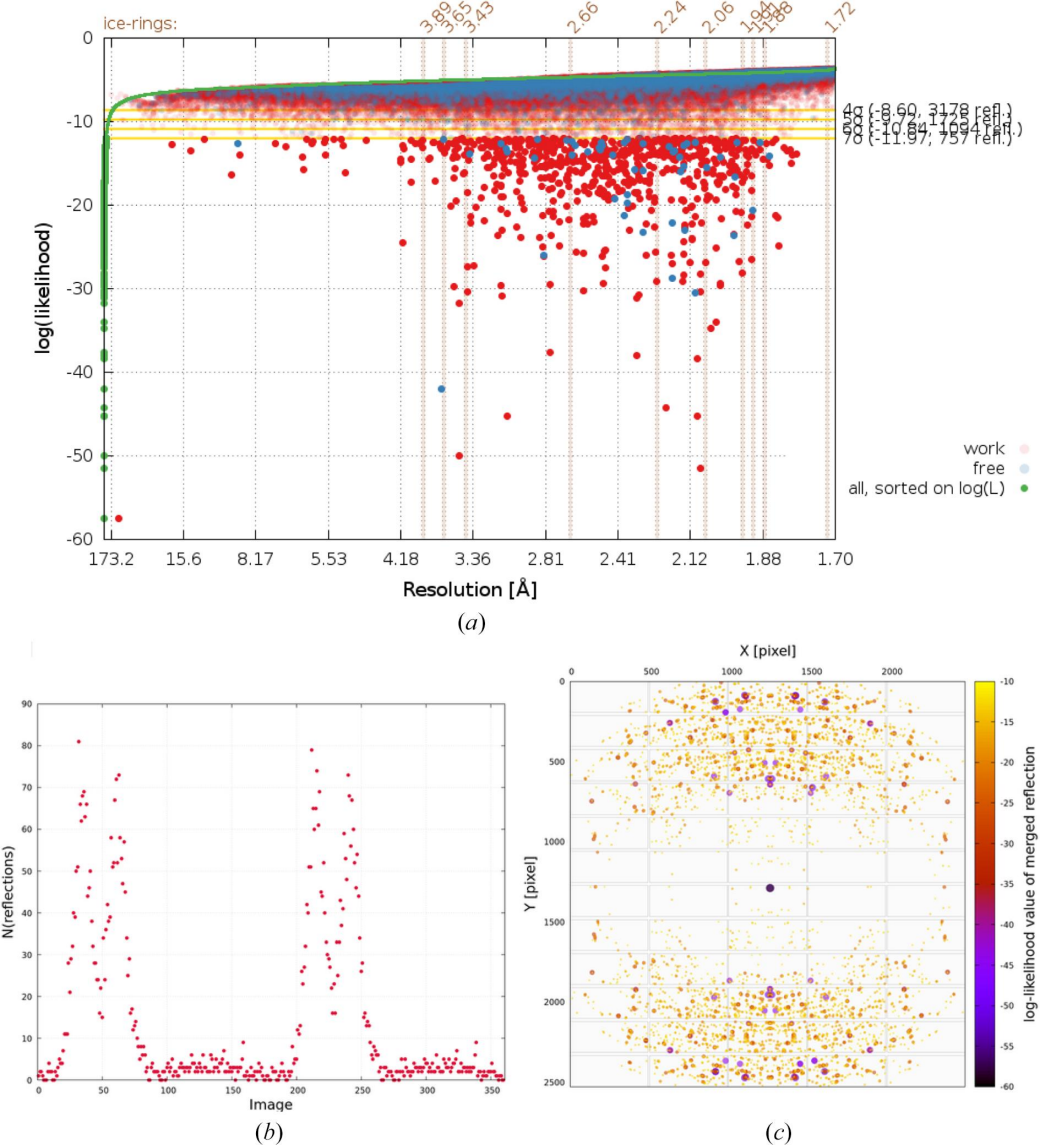
Analysis of poorly fitting reflection data during refinement (‘reflection auditing’) for PDB entry 6vzu. (*a*) Log(likelihood) values of each unique reflection after *BUSTER* refinement as a function of resolution: a large number of reflections with log(likelihood) values below −7σ are visible (working reflections are shown in red and test-set reflections in blue). (*b*) After determining the image number of all measurements contributing to those low-log(likelihood) unique reflections, the number of these potentially suspect measurements is given as a function of image number: two clear regions are visible around images 50 and 230. (*c*) The same set of low-log(likelihood) unique reflections is analysed to determine the detector coordinates of each contributing measurement and shown as a function of detector surface, with the image origin at the top left [circles coloured and scaled according to the log(likelihood) value of the merged unique reflection associated with these unmerged measurements].

After re-refinement using the deposited model and reflection data for PDB entry 6vzu (Mahalingan *et al.*, 2020 ▸), the analysis of per-reflection log(likelihood) values shows a surprisingly large number of outliers (below 7σ) that cover a wide resolution range (see Fig. 5 ▸). However, they do occur within two very distinct image ranges 180° apart from each other during data collection. The distribution of those outliers as a function of detector position shows that they lie far away from the horizontal spindle axis. Together, these two observations suggest a processing issue due to reflection overlap caused by too large a rotation range per image.

### 4.3. Radiation-damage analysis via *F*(early) − *F*(late) maps

#### 4.3.1. Radiation damage at the ligand

**Figure 6:**
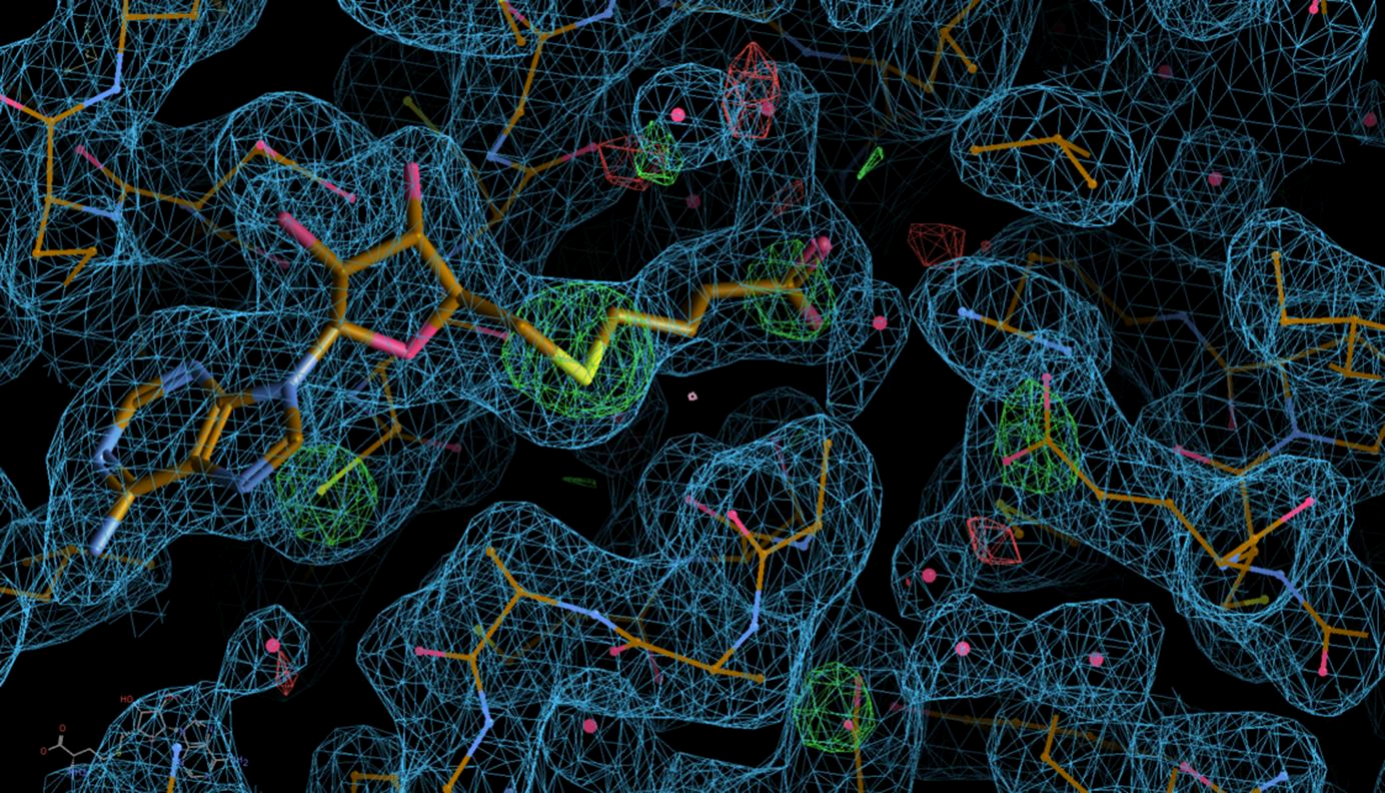
Model and maps after re-refinement of PDB entry 7zkg using *BUSTER* and the re-merged deposited data. The 2*mF*_o_ − *DF*_c_ electron density is shown in blue at 1 r.m.s. and the *F*(early) − *F*(late) difference map is shown in green (positive) and red (negative) at 5 r.m.s.. The *S*-adenosylhomocysteine (SAH) cofactor is shown in a thicker ball-and-stick presentation than the protein. Several typical indications of radiation damage are visible on the protein (from left to right): CysA64, AspA156 and GluA36. The cofactor itself seems to have suffered radiation damage to the sulfur and carboxyl group at the cysteine end.

The deposited data for PDB entry 7zkg (Amariei *et al.*, 2022 ▸) contain the scaled and unmerged reflection data that allow the generation of a set of *F*(early) and *F*(late) amplitudes (using the first 195 and last 219 images, respectively) in addition to the amplitudes after merging reflections from all 3600 images. After *BUSTER* refinement, the *F*(early) − *F*(late) difference map shows some very pronounced positive peaks (loss of electrons) for several Asp and Glu carboxyl groups. Furthermore, the *S*-adenosylhomocysteine (SAH) cofactor has additional indications of radiation damage, as can be seen in the *F*(early) − *F*(late) difference map (see Fig. 6 ▸).

#### 4.3.2. Improved model parametrization

PDB entry 5kco (Structural Genomics Consortium, unpublished work) contains an ‘early stage, low affinity fragment candidate modelled at reduced occupancy’: compound 6RO, *N*-(4-chlorophenyl)methanesulfonamide. This is modelled with an overall occupancy of 0.84 for all atoms, with the Cl atom showing a significantly higher *B* factor (32 Å^2^) than the other non-H atoms (18 Å^2^). A re-refinement of the deposited model against the deposited merged amplitudes using *BUSTER* results in final *R* and *R*_free_ values of 0.180 and 0.201, respectively, compared with the deposited values of 0.183 and 0.215 using *REFMAC* (Murshudov *et al.*, 2011 ▸). The occupancy of the 6RO compound refines to 0.73: this includes an automatic adjustment of the atomic scattering factor (real part) for Cl and S atoms for the given data-collection wavelength of 0.9282 Å. The *B* factor for the Cl atoms decreases slightly to a value of 26 Å^2^, which is still higher than the average for other non-H atoms (18 Å^2^). These values (an overall occupancy of around 0.73 and a chlorine *B* factor of about 30 Å^2^) do not change much if using either the deposited unmerged intensities (merging them without scaling using *AIMLESS* and calculating amplitudes using *TRUNCATE*; French & Wilson, 1978 ▸) or reprocessing the deposited raw diffraction data using *autoPROC*.

The data from this last step (reprocessing the raw diffraction data using *autoPROC*) provide additional opportunities for further analysis: not only do we have the (overall) average set of amplitudes using all 1500 images available, but also the *F*(early) and *F*(late) amplitudes based on images 1–717 and 1081–1500, respectively. The *F*(early) − *F*(late) map produced by *BUSTER* shows a very strong and clear peak at the Cl atom, suggesting significant radiation damage throughout the data collection (see Fig. 7 ▸).

*BUSTER* refinement against those overall amplitudes using a separate occupancy parameter for the Cl atom and for all other atoms results in *R* and *R*_free_ values of 0.185 and 0.207, respectively. The refined occupancies are 0.39 and 0.79 for the Cl and non-Cl atoms, respectively, while the respective *B* factors are now 20 and 19 Å^2^. This confirms that the average occupancy of the Cl atom over the exposure of the crystal to X-rays is lower by a factor of two compared with the rest of the molecule. The separate parametrization of occupancy for the radiation-sensitive and radiation-insensitive parts of the compound [as confirmed by these *F*(early) − *F*(late) features] not only prevents an artificial lowering of inadequate overall occupancy parameters, but also prevents the atomic isotropic *B* factor from becoming artificially inflated to compensate for the incorrect model parametrization.

Two additional refinements against the amplitudes from the ‘early’ and ‘late’ data sets confirm the effect of radiation damage on the Cl atom via refined occupancies of 0.46 and 0.27, respectively. This shows that even for the ‘early’ data set (94% completeness with a minimum of data used given the orientation of the crystal and the starting angle chosen) a significant amount of radiation damage is already detectable.

### 4.4. Outlook for the re-analysis of deposited unmerged data

Between 14 July 2022 and 13 July 2023, 3662 new PDB entries based on X-ray diffraction data were deposited. Of these, 44 (1.2%) contained unmerged reflection data with _diffrn_refln.pdbx_image_id data (recording the image number that a given unmerged reflection was measured on). 19 of these also contained the pixel coordinates for each observation on the detector surface (as the items _diffrn_refln.pdbx_detector_x and _diffrn_refln.pdbx_detector_y). These would allow additional analysis and remediation as described above directly on the deposited processed data. We hope that in the future more depositors take advantage of the possibility of depositing their scaled and unmerged reflection data containing these additional metadata.

## 5. Discussion

As we have seen from the examples given above, unmerged reflection data can carry a wealth of information that can be used to improve data quality as well as the resulting structural model and model parametrization, even if the latter is the result of standard crystallographic refinement against a set of merged amplitudes or intensities. In the same way that modern processing packages such as *autoPROC* employ various methods of iteration to improve integration and scaling through the analysis of outliers (for example via automatic ice-ring detection) or via the automatic exclusion of poor image ranges (using the per-image fitness analysis), model refinement and analysis against merged reflection data can also provide valuable feedback towards further improvements in data processing.

In our hands, the newly introduced per-image fitness analysis seems to be a robust metric for detecting poor image ranges at the data-processing stage. Using reflection auditing to correlate outliers in the merged data with the underlying unmerged data and ultimately with the actual raw data and the experimental setup employed can further highlight sometimes very subtle problems. The presented computation of *F*(early) − *F*(late) radiation-damage analysis maps should become a standard feature of any data-processing and refinement procedure whenever the type of experiment allows the generation of the required data sets from adequate subsets of images. 

## Figures and Tables

**Figure 7:**
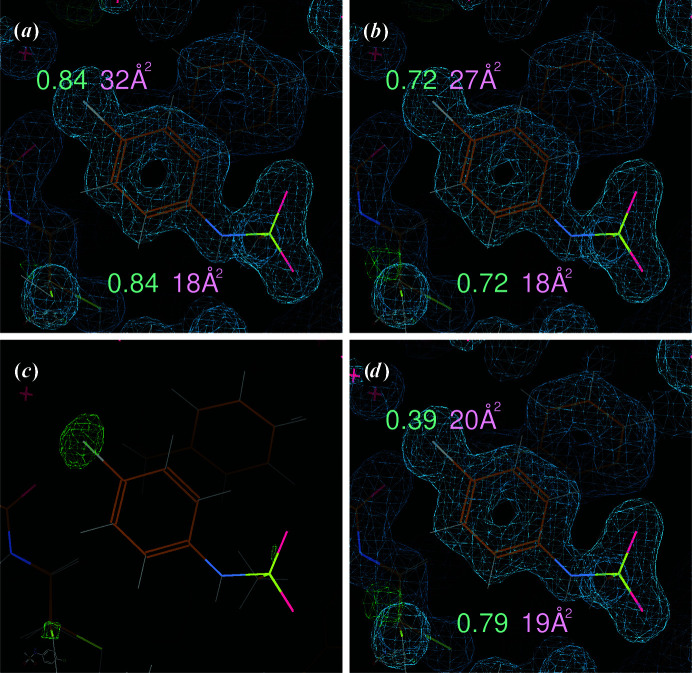
Electron-density and difference density maps for PDB entry 5kco after re-processing in *autoPROC* and re-refinement in *BUSTER*. The 6RO compound is shown with surrounding protein residues, a 2*mF*_o_ − *DF*_c_ density map at 1.0 r.m.s. and *mF*_o_ − *DF*_c_ difference density at 3.5 r.m.s.. The occupancy and isotropic *B* factor for the Cl atom are shown close to that atom, while those for the non-Cl atoms are shown below the compound. (*a*) Deposited model of 6RO in PDB entry 5kco. (*b*) After re-processing the raw diffraction data with *autoPROC* and refining a single occupancy over all compound atoms. (*c*) *F*(early) − *F*(late) map at 5.0 r.m.s. showing a strong peak at the Cl atom. (*d*) After using separate occupancy parameters for the Cl and non-Cl compound atoms.

**Table 1:** Data-quality metrics for deposited unmerged reflection data for PDB entry 8aj2 before and after exclusion of poor image ranges using fitness analysis

	Overall (46.16–2.20 Å)					Outer shell (2.27–2.20 Å)				
		All data†		Selected‡			All data†		Selected‡	
	Deposited§	As-is¶	Rescaled††	As-is¶	Rescaled††	Deposited§	As-is¶	Rescaled††	As-is¶	Rescaled††
*R*_merge_ ‡‡	0.071	0.071	0.073	0.058	0.059	1.143	1.139	1.152	0.865	0.871
*R*_meas_ §§	0.076	0.075	0.077	0.062	0.063	1.196	1.193	1.206	0.925	0.932
*R*_p.i.m._ ¶¶	0.022	0.022	0.023	0.022	0.023	0.352	0.351	0.355	0.327	0.330
Total No. of unique reflections	28787	28761		28749		2454	2559		2553	
〈*I*/σ(*I*)〉†††	18.7	18.8	17.1	16.7	17.0	2.1	2.1		2.1	
Completeness (%)	99.9	100.0		99.9		99.4	99.6		99.3	
Multiplicity	11.3	11.3		7.9		11.4	11.4		7.9	
CC_1/2_ [Table-fn tfn10]	0.990	0.999		0.999		0.751	0.778	0.785	0.790	0.792

† Using deposited unmerged reflection data for all 2000 images.

‡ Using deposited unmerged reflection data for the first 1393 images only.

§ Data-quality metrics given in the archived PDBx/mmCIF entry for PDB entry 8aj2.

¶ Using deposited unmerged data without any further scaling or error model adjustment.

†† Using deposited unmerged data for scaling and error model adjustment in *AIMLESS*.

‡‡ *R*_merge_ = 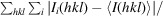 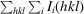 (Evans, 2006 ▸; Einspahr & Weiss, 2012 ▸).

§§ *R*_meas_ = 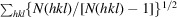 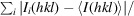 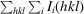 (Diederichs & Karplus, 1997 ▸; Weiss & Hilgenfeld, 1997 ▸).

¶¶ *R*_p.i.m._ = 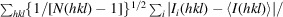 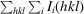 (Weiss, 2001 ▸).

††† Average (within given resolution limits) of inverse-variance-weighted mean intensities over their corresponding error.

‡‡‡ Correlation coefficient between two randomly chosen ‘half’ sets (Karplus & Diederichs, 2012 ▸; Evans, 2011 ▸).

## Footnotes

1 Since the 24 February 2021 release.

2 This feature has been available since the 16 July 2021 release.

3 Available since the 14 December 2015 releases of these two packages.

## References

[bb1] Agirre, J., Atanasova, M., Bagdonas, H., Ballard, C. B., Baslé, A., Beilsten-Edmands, J., Borges, R. J., Brown, D. G., Burgos-Mármol, J. J., Berrisford, J. M., Bond, P. S., Caballero, I., Catapano, L., Chojnowski, G., Cook, A. G., Cowtan, K. D., Croll, T. I., Debreczeni, J. É., Devenish, N. E., Dodson, E. J., Drevon, T. R., Emsley, P., Evans, G., Evans, P. R., Fando, M., Foadi, J., Fuentes-Montero, L., Garman, E. F., Gerstel, M., Gildea, R. J., Hatti, K., Hekkelman, M. L., Heuser, P., Hoh, S. W., Hough, M. A., Jenkins, H. T., Jiménez, E., Joosten, R. P., Keegan, R. M., Keep, N., Krissinel, E. B., Kolenko, P., Kovalevskiy, O., Lamzin, V. S., Lawson, D. M., Lebedev, A. A., Leslie, A. G. W., Lohkamp, B., Long, F., Malý, M., McCoy, A. J., McNicholas, S. J., Medina, A., Millán, C., Murray, J. W., Murshudov, G. N., Nicholls, R. A., Noble, M. E. M., Oeffner, R., Pannu, N. S., Parkhurst, J. M., Pearce, N., Pereira, J., Perrakis, A., Powell, H. R., Read, R. J., Rigden, D. J., Rochira, W., Sammito, M., Sánchez Rodríguez, F., Sheldrick, G. M., Shelley, K. L., Simkovic, F., Simpkin, A. J., Skubak, P., Sobolev, E., Steiner, R. A., Stevenson, K., Tews, I., Thomas, J. M. H., Thorn, A., Valls, J. T., Uski, V., Usón, I., Vagin, A., Velankar, S., Vollmar, M., Walden, H., Waterman, D., Wilson, K. S., Winn, M. D., Winter, G., Wojdyr, M. & Yamashita, K. (2023). *Acta Cryst.* D**79**, 449–461.

[bb3] Amariei, D. A., Pozhydaieva, N., David, B., Schneider, P., Classen, T., Gohlke, H., Weiergräber, O. H. & Pietruszka, J. (2022). *ACS Catal.* **12**, 14130–14139. 10.1021/acscatal.3c04952 PMC10775177 38205025

[bb4] Assmann, G., Brehm, W. & Diederichs, K. (2016). *J. Appl. Cryst.* **49**, 1021–1028. 10.1107/S1600576716005471 PMC4886987 27275144

[bb5] Batista, M., Donker, E. I., Bon, C., Guillien, M., Caisso, A., Mourey, L., François, J.-M., Maveyraud, L. & Zerbib, D. (2023). *J. Mol. Biol.* **435**, 168048. 10.1016/j.jmb.2023.168048 36933821

[bb6] Bricogne, G., Blanc, E., Brandl, M., Flensburg, C., Keller, P., Paciorek, W., Roversi, P., Sharff, A., Smart, O. S., Vonrhein, C. & Womack, T. (2023). *BUSTER*, version 2.10.4. Global Phasing Ltd, Cambridge, United Kingdom.

[bb8] Diederichs, K. (2006). *Acta Cryst.* D**62**, 96–101. 10.1107/S0907444905031537 16369098

[bb9] Diederichs, K. & Karplus, P. A. (1997). *Nat. Struct. Mol. Biol.* **4**, 269–275. 10.1038/nsb0497-269 9095194

[bb10] Diederichs, K. & Karplus, P. A. (2013). *Acta Cryst.* D**69**, 1215–1222. 10.1107/S0907444913001121 PMC3689524 23793147

[bb11] Douse, C. H., Bloor, S., Liu, Y., Shamin, M., Tchasovnikarova, I. A., Timms, R. T., Lehner, P. J. & Modis, Y. (2018). *Nat. Commun.* **9**, 651. 10.1038/s41467-018-03045-x PMC5811534 29440755

[bb12] Einspahr, H. & Weiss, M. (2012). *International Tables for Crystallo­graphy*, Vol. F, pp. 64–74. Dordrecht: Springer.

[bb13] Emsley, P., Lohkamp, B., Scott, W. G. & Cowtan, K. (2010). *Acta Cryst.* D**66**, 486–501. 10.1107/S0907444910007493 PMC2852313 20383002

[bb14] Evans, P. (2006). *Acta Cryst.* D**62**, 72–82. 10.1107/S0907444905036693 16369096

[bb15] Evans, P. R. (2011). *Acta Cryst.* D**67**, 282–292. 10.1107/S090744491003982X PMC3069743 21460446

[bb16] Evans, P. R. & Murshudov, G. N. (2013). *Acta Cryst.* D**69**, 1204–1214. 10.1107/S0907444913000061 PMC3689523 23793146

[bb17] French, S. & Wilson, K. (1978). *Acta Cryst.* A**34**, 517–525.

[bb7] Gahbauer, S., Correy, G. J., Schuller, M., Ferla, M. P., Doruk, Y. U., Rachman, M., Wu, T., Diolaiti, M., Wang, S., Neitz, R. J., Fearon, D., Radchenko, D. S., Moroz, Y. S., Irwin, J. J., Renslo, A. R., Taylor, J. C., Gestwicki, J. E., von Delft, F., Ashworth, A., Ahel, I., Shoichet, B. K. & Fraser, J. S. (2023). *Proc. Natl Acad. Sci. USA*, **120**, e2212931120. 10.1073/pnas.2212931120 PMC9926234 36598939

[bb18] Garman, E. F. & Weik, M. (2023). *Curr. Opin. Struct. Biol.* **82**, 102662. 10.1016/j.sbi.2023.102662 37573816

[bb20] Kabsch, W. (2010). *Acta Cryst.* D**66**, 125–132. 10.1107/S0907444909047337 PMC2815665 20124692

[bb21] Karplus, P. A. & Diederichs, K. (2012). *Science*, **336**, 1030–1033. 10.1126/science.1218231 PMC3457925 22628654

[bb22] Mahalingan, K. K., Keenan, E. K., Strickland, M., Li, Y., Liu, Y., Ball, H. L., Tanner, M. E., Tjandra, N. & Roll-Mecak, A. (2020). *Nat. Struct. Mol. Biol.* **27**, 802–813. 10.1038/s41594-020-0462-0 32747782

[bb23] Mueller, M., Wang, M. & Schulze-Briese, C. (2012). *Acta Cryst.* D**68**, 42–56. 10.1107/S0907444911049833 PMC3245722 22194332

[bb24] Murshudov, G. N., Skubák, P., Lebedev, A. A., Pannu, N. S., Steiner, R. A., Nicholls, R. A., Winn, M. D., Long, F. & Vagin, A. A. (2011). *Acta Cryst.* D**67**, 355–367. 10.1107/S0907444911001314 PMC3069751 21460454

[bb26] Sanctis, D. de & Nanao, M. H. (2012). *Acta Cryst.* D**68**, 1152–1162. 10.1107/S0907444912023475 22948916

[bb27] Schiltz, M., Dumas, P., Ennifar, E., Flensburg, C., Paciorek, W., Vonrhein, C. & Bricogne, G. (2004). *Acta Cryst.* D**60**, 1024–1031. 10.1107/S0907444904006377 15159561

[bb28] Sharff, A., Keller, P., Vonrhein, C., Smart, O. S., Flensburg, C., Paciorek, W., Tickle, I., Fogh, R., Wojdyr, M. & Bricogne, G. (2023). *Pipedream*, version 1.4.0. Global Phasing Ltd, Cambridge, United Kingdom.

[bb29] Sharma, D., Singh, M., Kaur, P. & Das, U. (2022). *Acta Cryst.* D**78**, 835–845. 10.1107/S2059798322005290 35775983

[bb31] Terwilliger, T. C. & Bricogne, G. (2014). *Acta Cryst.* D**70**, 2533–2543. 10.1107/S1399004714017040 PMC4188001 25286839

[bb32] Thorn, A., Parkhurst, J., Emsley, P., Nicholls, R. A., Vollmar, M., Evans, G. & Murshudov, G. N. (2017). *Acta Cryst.* D**73**, 729–737. 10.1107/S205979831700969X PMC5586246 28876236

[bb33] Vonrhein, C., Flensburg, C., Keller, P., Sharff, A., Smart, O., Paciorek, W., Womack, T. & Bricogne, G. (2011). *Acta Cryst.* D**67**, 293–302. 10.1107/S0907444911007773 PMC3069744 21460447

[bb34] Weiss, M. S. (2001). *J. Appl. Cryst.* **34**, 130–135.

[bb35] Weiss, M. S. & Hilgenfeld, R. (1997). *J. Appl. Cryst.* **30**, 203–205.

[bb36] Westbrook, J. D., Young, J. Y., Shao, C., Feng, Z., Guranovic, V., Lawson, C. L., Vallat, B., Adams, P. D., Berrisford, J. M., Bricogne, G., Diederichs, K., Joosten, R. P., Keller, P., Moriarty, N. W., Sobolev, O. V., Velankar, S., Vonrhein, C., Waterman, D. G., Kurisu, G., Berman, H. M., Burley, S. K. & Peisach, E. (2022). *J. Mol. Biol.* **434**, 167599. 10.1016/j.jmb.2022.167599 PMC10292674 35460671

[bb38] Williams, T. & Kelley, C. (2014). *Gnuplot 4.6: An Interactive Plotting Program.* http://www.gnuplot.info/docs_4.6/gnuplot.pdf.

[bb39] wwPDB (2007). *Announcement: Experimental Data will be Required for Depositions Starting February 1, 2008.* https://www.wwpdb.org/news/news?year=2007#5764490799cccf749a90cd7f.

[bb40] wwPDB (2021). *Improved Support for Extended PDBx/mmCIF Structure Factor Files.* https://www.wwpdb.org/news/news?year=2021#60638da1931d5660393084c3.

[bb25] wwPDB Consortium (2019). *Nucleic Acids Res.* **47**, D520–D528. 10.1093/nar/gky949 PMC6324056 30357364

